# Supplementary material for: Pretreatment with Zonisamide Mitigates Oxaliplatin-Induced Toxicity in Rat DRG Neurons and DRG Neuron–Schwann Cell Co-Cultures
Source: Int J Mol Sci. 2022 Sep 1;23(17):9983. doi: 10.3390/ijms23179983 (PMC9456039; doi:10.3390/ijms23179983)
Supplement: Supplementary file 1 [file ijms-23-09983-s001.zip › ijms-1870184-supplementary.pdf]

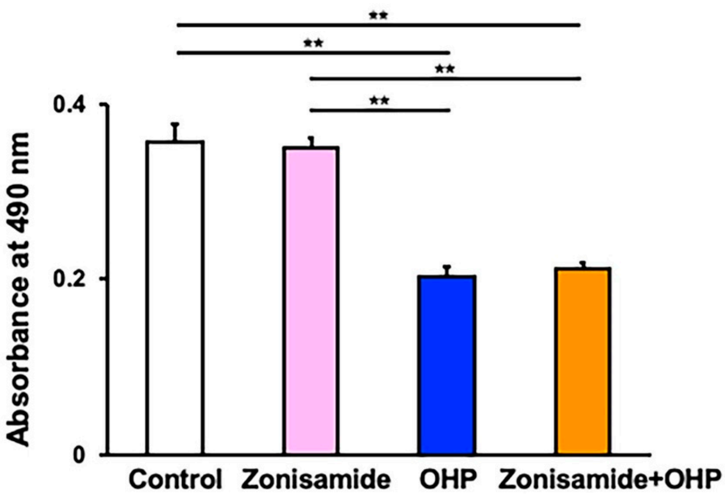

Supplementary Figure S1. Preincubation of zonisamide (100 μM) for 6 h fails to mitigate the cell death caused as a result of exposure to OHP (75 μM) for 24 h. Data are presented as the mean ± *SD* of 6-9 experiments, respectively; **: $p < 0.01$ (as defined by Tukey–Kramer).
